# A Single Center Case Series of Gender-Affirming Surgeries and the Evolution of a Specialty Anesthesia Team

**DOI:** 10.3390/jcm11071943

**Published:** 2022-03-31

**Authors:** Nelson J. Aquino, Elizabeth R. Boskey, Steven J. Staffa, Oren Ganor, Alyson W. Crest, Kristin V. Gemmill, Joseph P. Cravero, Bistra Vlassakova

**Affiliations:** 1 Department of Anesthesiology, Critical Care and Pain Medicine, Boston Children’s Hospital, Boston, MA 02115, USA; steven.staffa@childrens.harvard.edu (S.J.S.); alyson.crest@childrens.harvard.edu (A.W.C.); kristin.gemmill@childrens.harvard.edu (K.V.G.); joseph.cravero@childrens.harvard.edu (J.P.C.); bistra.vlassakova@childrens.harvard.edu (B.V.); 2 Center for Gender Surgery, Boston Children’s Hospital, Boston, MA 02115, USA; elizabeth.boskey@childrens.harvard.edu (E.R.B.); oren.ganor@childrens.harvard.edu (O.G.)

**Keywords:** transgender, gender affirmation surgery, anesthesia, gender dysphoria, chest reconstruction, genital surgery

## Abstract

Most minors and young transgender persons wishing to undergo gender-affirming surgery need to seek specialists affiliated with gender affirmation programs in adult hospitals. Research suggests gender affirmation surgery has been established as an effective and medically indicated treatment for gender dysphoria. Although most data on gender-affirming surgeries are from adult populations, there is growing literature establishing their effectiveness in adolescents and young adults. Therefore, it is critical to evaluate the perioperative outcomes for gender-diverse youth to deliver safe and affirming care. The primary objective of this retrospective case series is to examine the perioperative characteristics and outcomes of patients with gender identity disorders (International Classification of Diseases [ICD]-10-code F64) who underwent chest reconstruction (mastectomy) and genital surgery (phalloplasty, metoidioplasty, and vaginoplasty) in a pediatric academic hospital. The secondary aim is to evaluate the value of a specialized anesthesia team for improving clinical outcomes, interdisciplinary communication, and further advancing the transgender perioperative experience. We identified 204 gender affirmation surgical cases, 177 chests/top surgeries, and 27 genital/bottom surgeries. These findings indicate gender-diverse individuals who underwent life-changing surgery at our institution had a median age of 18 years old, with many patients identifying as transmen. Our data suggests that postoperative pain was significant, but adverse events were minimal. The evolution of a specialty anesthesia team and initiatives (anesthesia management guidelines, scheduling, continuity, and education) necessitate direct care coordination and multidisciplinary planning for gender affirmation surgery in transgender youth.

## 1. Introduction

Transgender and gender non-binary individuals are a growing demographic worldwide (See Appendix A for glossary of terms). An estimated 0.7% of the youth in the United States (ages 13–17) identify as transgender [1]. Over the last decade, access to gender-affirming specialized surgical care has increased among adolescents and young adults. Gender-affirmation surgery has been established as an effective and medically indicated treatment for gender dysphoria [2]. Although most data on gender-affirming surgeries are from adult populations, there is growing literature establishing their effectiveness in adolescents and young adults [3].

The protection of transgender rights and the acknowledgement of gender affirmation medical care appear to have allowed for significant paradigm shifts in surgical access for transgender adolescents, although these improvements are not universal [4].

Until recently, most minors and young transgender persons wishing to undergo gender-affirming surgery needed to seek specialists affiliated with adult hospitals. In the United States, there is growing support for improved access to gender-affirming surgical care at pediatric institutions. The World Professional Association of Transgender Health (WPATH) is an interdisciplinary body of health professionals that establishes Standards of Care (WPATH SOC) to advance transgender healthcare and surgeries through evidence-based medicine. The international consensus of WPATH SOC guidelines establish procedural safeguards to protect patients, families, and healthcare professionals caring for transgender and gender-diverse persons [5]. The Center for Gender Surgery (CfGS) at Boston Children’s Hospital (BCH) was the first pediatric center in the United States to offer gender-affirming chest surgeries for individuals over 15 years old and genital surgeries for those over 17 years of age. In the four years since its inception, CfGS has completed over 300 gender-affirming surgeries.

The primary objective of this case series was to examine the patient characteristics, specific surgical techniques, and perioperative outcomes of masculinizing chest reconstruction and genital surgeries in a pediatric academic hospital. The secondary aim of the case series was to describe the evolution of a specialized anesthesia care team model known as the Gender Affirming Surgical Perioperative Program (GASPP).

## 2. Materials and Methods

This study was approved by the institutional review board (IRB) of Boston Children’s Hospital, and the requirement for written informed consent was waived by the IRB. A retrospective case series analysis was performed to collect comprehensive data on all chest reconstruction and genital surgeries from January 2017 to August 2020. Boston Children’s Hospital is a pediatric academic hospital in the United States, providing gender affirmation surgery to patients aged 15 years to 35 years of age, including gender-affirming primary and multispecialty care involving inpatient and outpatient care.

Patients included in the study were identified by the International Classification of Diseases (ICD)-10-code F64 for gender identity disorders. The following Current Procedural Terminology (CPT) codes were included: chest reconstruction procedures, vaginectomy and phalloplasty procedures, vaginoplasty surgeries, and metoidioplasty surgical procedures. This led to a total of 204 surgical cases included in this case series.

Data was extracted from patient electronic medical records, anesthesia databases, and billing records stratified by surgical procedure type. Demographic data collected included age, weight, the American Society of Anesthesiologists Physical Status (ASA-PS) classification, and gender identity. Perioperative characteristics identified were discharge plan, intensive care unit (ICU) admission, documented adverse events (hematoma and airway event), readmission, and hospital length of stay. Post-anesthesia care unit (PACU) data on emesis and pain scores (numerical pain rating scale [NPRS]) were collected, and inpatient data on opioid equivalents and postoperative nausea and vomiting (PONV) were identified.

Continuous variables (age, weight, opioid equivalents (based on morphine), length of stay, pain scores) were described using medians with accompanying full ranges, and categorical data (minor at time of surgery, ASA-PS, gender identity, age, weight, epidural, PONV, PACU pain, discharge plan, ICU admission, adverse events, and readmission) were presented as frequencies and percentages. Missing data were denoted by different denominators presented within subgroups for categorical variables. Descriptive statistics of the case series were presented overall for all cases, and separately for chest reconstructions and genital surgeries. Descriptive statistics were obtained using Stata (version 16.0, StataCorp LLC., College Station, TX, USA).

## 3. Results

### 3.1. Demographics and Patient Characteristics

Over the 3-year study period, a total of 204 gender affirmation surgical cases were identified: 177 chest/top and 27 genital/bottom surgeries (Table 1). Most cases were masculinizing chest reconstructions 177/204 (86.8%) with 65/177 (36.7%) of those patients being less than 18 years of age. Patient characteristics included a median age of 18 years old, with the overwhelming majority of the patients (90.7%) identifying as transmen. Ninety-five percent of patients presenting for gender reassignment surgeries were healthy American Society of Anesthesiologists physical status (ASA-PS) I and II.

### 3.2. Chest Reconstruction Data

One of the most regularly requested surgical treatments for transmasculine and non-binary patients is masculinizing chest reconstruction, also known as “top surgery” and formally as “mastectomy” [6]. The operative goals for chest reconstruction surgery involve producing a masculine chest contour by removing excess breast tissue and altering the nipple and areola with minimal chest wall scars [7]. For transmasculine gender-diverse youth, the existence of breast tissue and its growth during puberty can be highly upsetting and cause further gender dysphoria [8]. The anesthesia management goals for chest reconstruction surgery are to mitigate PONV, decrease opioid consumption, and prevent the formation of postoperative hematomas.

The demographic and patient characteristics of chest reconstruction patients are summarized in Table 2. There were a total of 177 chest reconstructions performed from January 2017 to August 2020. Patients coming for chest reconstruction surgery identified themselves mostly as trans masculine (92.1%) and non-binary (4.5%). The median age of patients was 18 with the youngest patient presenting at 15 years old. The rate of same-day discharge to home was 18.6% compared to floor at 81.4%. Median hospital length of stay in the chest reconstructions was 1.1 days. Inpatient opioid equivalents at 24 h represented a median of 0.4 mg/kg of morphine. Most patients presented to the post-anesthesia care unit (PACU) with low pain scores (64.4%). Rates of inpatient postoperative nausea and vomiting (PONV) were higher at 11.3% versus 1.1% in the post-anesthesia care unit (PACU). Eight adverse events were observed in the chest reconstruction cohort, consisting of 7 hematomas (4%) and one airway event (0.6%). The readmission rate in this cohort was 2.8% (n = 5).

### 3.3. Genital Surgeries Results

Typical forms of genital reconstruction (bottom surgery) include phalloplasty and metoidioplasty procedure for transmasculine patients and vaginoplasty for transfeminine patients. For transmasculine patients, the surgical goals of bottom surgery depend on patient specific desires. These include the removal of female genitalia, the formation of male genitalia, standing to void, the consolidation of erogenous feeling to the phallus, and future erectile prothesis and testicular implants [7]. The more extensive phalloplasty procedures consist of several steps, including vaginectomy, urethral lengthening, scrotoplasty, and creation of the neophallus, which is most commonly performed with a free tissue transfer from the radial forearm [7]. For transfeminine patients, the vaginoplasty procedure creates a sensate clitoris, vulva, and (optional) neovaginal canal to align with their gender identity. The anesthesia considerations for genital surgeries focus on preoperative anxiety, fluid management, pain management strategies (epidural anesthesia for vaginoplasty), and positioning as extended operating times present an increased risk of peripheral neuropathies to both upper and lower extremities.

Twenty-seven genital surgeries were performed and Table 3 summarizes the breakdown of cases. Epidural anesthesia was used in 4 vaginectomy cases, 0 phalloplasty cases, 5 vaginoplasty cases, 1 combined vaginectomy and phalloplasty case, and 1 combined vaginectomy and metoidioplasty case. Inpatient opioid equivalents at 24 h were highest in phalloplasty cases (median = 1.5 mg/kg) in comparison with 0.53 mg/kg for vaginectomies, 0.57 mg/kg for combined vaginectomy and phalloplasty, and 0.09 mg/kg for vaginoplasty. In phalloplasty cases, increased opioid equivalents in the absence of epidural anesthesia may be attributed to pain associated with the forearm flap. Among the combined vaginectomy and phalloplasty cases identified, two unplanned admissions within 48 h were noted. One for arm pain concerns and the other for uncontrolled pain. In the PACU, low pain scores (0–3) were observed in 3/9 (33.3%) of vaginectomies, 5/5 (100%) of vaginoplasties, and 1/1 (100%) of metoidioplasties. All other genital surgeries had planned intensive care unit (ICU) admissions to monitor the urethral anastomosis.

## 4. Discussion

In this study, we present the first descriptive anesthesia case series of transgender youth undergoing gender affirmation surgery. From our study, we describe establishment of an anesthesia care service designed to meet the specific needs of gender-diverse youth undergoing gender reassignment surgery.

### 4.1. Development of GASPP

Our perioperative services have seen an increasing number of transgender patients scheduled for procedures (gender affirming and routine). These patients present with significant complexities in the perioperative period (Table 4). In response to the clinical needs presented by this population, in November 2019, a core team of pediatric anesthesiologists, certified registered nurse anesthetists (CRNAs), an administrative lead, and a research nurse formally established the Gender Affirming Surgical Perioperative Program (GASPP). The primary objective of the GASPP was to appropriately address the preoperative (e.g., anxiety and comorbidities), intraoperative (e.g., unique anesthesia considerations), and postoperative needs (e.g., pain management and outcomes) of this unique patient population. The additional objectives of the GASPP were to provide welcoming and culturally sensitive care for gender-diverse patients and their families, with a consistent team of providers.

**Figure 1:**
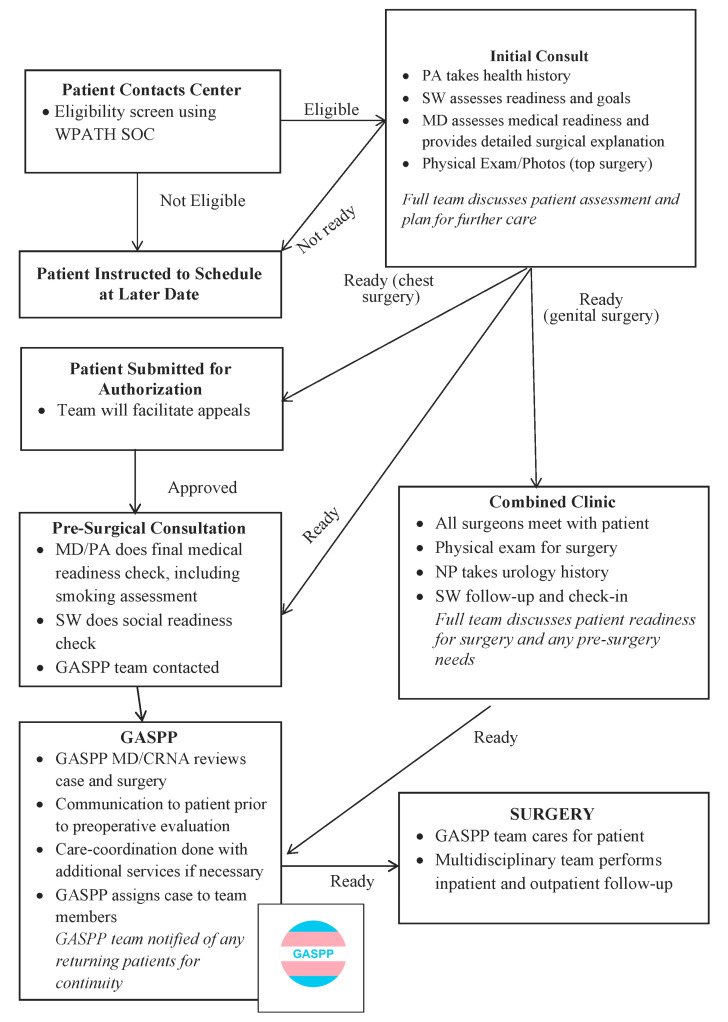
Center for Gender Surgery Patient Care Flow Chart for Chest and Genital Surgeries. WPATH SOC, World Professional Association of Transgender Health Standards of Care; PA, Physician Assistant; SW, Social Worker, MD, Medical Doctor; NP, Nurse Practitioner; CRNA: Certified Registered Nurse Anesthetist; GASPP, Gender Affirming Surgical Perioperative Program.

In addition to emotional and behavioral issues, transgender patients frequently have co-existing morbidities such as obesity, substance abuse, and congenital disorders, which could affect surgical planning and outcomes [9]. Prior to the development of GASPP, patients first met with their anesthesia and perioperative team on the day of surgery for a routinely scheduled procedure. The GASPP team recognized that these patients required more extensive preoperative evaluation and preparation, acknowledged the need for a dedicated anesthesia team for surgical planning, and initiated a partnership with the Center for Gender Surgery (CfGS) to participate in care coordination. (Figure 1) [4]. The creation of the GASPP team also fulfilled the purpose of supporting the institution’s mission of comprehensive and affirming health equity for all [4].

Beginning in June 2019, a GASPP attending anesthesiologist and CRNA were pre-assigned to each gender-affirming procedure to facilitate a multidisciplinary approach to patient care, and in-depth planning and communication. The goal was to provide consistent care through multi-staged procedures. The care coordination and anesthesia team-based approach was formulated after the Pediatric Perioperative Surgical Home model [15]. Furthermore, an official GASPP email was created to offer clinicians, patients, and their families direct communication. This communication channel allows for individualized care planning between primary care, surgery, anesthesia, nursing, the patient, and family.

### 4.2. Initiatives of GASPP

The GASPP team instituted several initiatives to improve the transgender perioperative experience (Table 5). Anesthesia management guidelines were created for each surgical procedure with the intent to improve clinical outcomes, decrease adverse events, and improve patient and team satisfaction. Anesthesia guidelines for chest reconstruction, phalloplasty, and vaginoplasty procedures were developed by applying evidenced-based anesthesia principles and experiential knowledge gained from gender affirmation cases. Capturing all the elective gender-affirming procedures from 2019–2020, GASPP members continuously reviewed the data to tailor and update anesthesia management to improve perioperative outcomes and adapt to changing surgical techniques. The clinical pearls are posted on the departmental internal website and are used as a management guideline by all anesthesia providers.

The benefits of working with a gender-focused, dedicated anesthesia team extend far beyond the experiences of each individual patient. Multidisciplinary collaboration, continuity of care, and application of evidence-based care principles of pediatric anesthesia practice are fundamental to the success of the GASPP. McIntosh [16] advocates that healthcare providers in interdisciplinary teams who communicate effectively in the complex care of transgender patients help to reduce gender dysphoria and produce improved outcomes and patient care experiences [16].

Through a careful process of outcome evaluation, collaboration with surgeons, and consultation with perioperative nursing teams, we enhanced and codified our individualized anesthesia considerations for chest and genital surgeries. There is a growing literature comparing Enhanced Recovery After Anesthesia (ERAS) pathways in adult surgical procedures, and a few reports of ERAS protocols are emerging to streamline pediatric perioperative care and improve patient outcomes. In a review and meta-analysis of Enhanced Recovery After Anesthesia (ERAS) pathways of adult breast reconstruction patients, the evidence supported the reduction in postoperative opioid use and a decrease in length of hospital stay with a mean difference of 1.7 days compared to traditional care [17]. In a retrospective study of 57 female-to-male transgender adult patients undergoing chest contouring surgery, 84.2% were discharged to home within 24 h after their procedure [18]. In our chest reconstruction anesthesia guideline, we recommend a combination of antiemetic medications (scopolamine patch, corticosteroid, and 5-HT3 antagonist) and intraoperative intravenous infusions of propofol to minimize PONV. Persing et al. [19] recommend multimodal PONV prophylaxis and total intravenous anesthesia (TIVA) techniques in adult ERAS pathways for breast reconstruction to mitigate risk of nausea and vomiting [19]. Our findings after chest reconstruction surgery are aligned with the findings in the adult literature. Creation of this service and implementation of population-specific anesthesia protocols may decrease opioid requirements and postoperative nausea and vomiting.

Adolescents who identify as transgender and gender nonconforming experience a high rate of stigma in the hospital environment and face inequitable access to primary care and mental health resources compared to their cisgender peers [20]. Severe anxiety, trauma responses, suicidal ideation, and self-harm, in addition to experience-based fears of healthcare discrimination, are only a few of the challenges faced by patients [2,21,22]. This complicated psychological constellation can be quite challenging for health providers unfamiliar with this population. Unintended misidentification such as misgendering can contribute to patient anxiety and dissatisfaction, as well as creating provider discomfort [2]. The creation of our GASPP allowed us to maximize the awareness of these issues among the providers caring for these patients and may well have added to the positive outcomes we report.

Gender-affirming surgery has been shown to improve quality of life. Although current research has been focused mostly on adults, a study of 136 youth demonstrated significant improvements in chest dysphoria in transmasculine individuals undergoing chest reconstruction [8]. A prospective study of 190 transfeminine adults undergoing gender affirmation surgery found that their short-term data support an improved quality of life after gender affirming surgery, although long-term data was still needed [23]. A systematic review of seven studies involving 420 persons (259 transfeminine and 122 transmasculine individuals) on quality of life (QOL) data in adults reported transgender individuals generally had improvements in body image and other areas associated with quality of life after gender-affirming surgery [24]. Implementation of Patient-Reported Outcome Measures is reported in the literature to assess subjective outcomes for top and bottom surgery. However, more studies are needed to validate patient satisfaction surveys and instruments on quality of life after gender affirmation surgery in adults [25,26,27]. Researchers are currently working on developing a comprehensive patient reported outcome measure for evaluating gender-affirming treatments—the GENDER-Q [28].

Care coordination between anesthesia and surgical staff has allowed GASPP team members to anticipate and plan for surgeries well in advance. As more gender-diverse youth require general anesthesia for dental, orthopedic, and other non-gender-affirming procedures, the GASPP team can offer continuity of care that fosters a milieu of trust and familiarity with their transition journey. The study by Kattari et al. [29] on transgender-inclusive providers and mental health outcomes show gender-diverse patients having a transgender-inclusive provider were less likely to report symptoms of depression, anxiety, and suicide, compared to those who did not report having a transgender-inclusive provider.

There were several limitations in our study. It was a retrospective case series based on multiple anesthesia records and patient medical charts subject to variability on individual documentation practice and limitations of what data is routinely recorded. All records were manually audited to confirm the correctness of the perioperative, PACU, inpatient data, including gender identity, diagnosis, type of surgery, medications, and adverse events within the Anesthesia Information Management System (AIMS) record and medical records. The gender-affirming procedures were performed in a single center at our main hospital and satellite surgery center. Surgical techniques for genital procedures varied depending on the surgeons’ desired method and evolved over the course of this study.

Future studies are needed to compare various anesthesia techniques for optimizing outcomes after different procedures for this population. Creation of this service and implementing population-specific anesthesia protocols may decrease opioid requirements and postoperative nausea and vomiting. The GASPP team is in the process of adapting anesthesia guidelines into formal Enhanced Recovery After Anesthesia (ERAS) protocols and working to develop prospective studies to follow perioperative anxiety, patient satisfaction, and quality of life for transgender children and adolescents.

## 5. Conclusions

The collaboration between the GASPP and CfGS offered gender-diverse youth access to comprehensive, developmentally appropriate surgical care to meet their physical, mental, emotional, and spiritual needs that spans the entire consultation, perioperative, and postoperative period. Furthermore, the dedicated team approach supported improved perioperative care and outcomes. However, the reproducibility of this approach needs further studies to be proven. As more pediatric institutions develop their gender-affirming surgical centers, we present this model as a guide for pediatric anesthesia departments.

## Figures and Tables

**Table 1:** Demographics and Patient Characteristics.

Variable	Median (Range) or n (%)
Number of Cases	204
Age (years)	18 (15, 34)
Patient a Minor on Date of Surgery	65 (31.9%)
Weight (kg)	70.8 (44.3, 141.4)
**American Society of Anesthesiologists Physical Status**	
I	46 (22.6%)
II	148 (72.6%)
III	10 (4.9%)
**Gender Identity**	
Trans Man	185 (90.7%)
Trans Woman	10 (4.9%)
Non-binary	8 (3.9%)
Cis Female	1 (0.5%)
**Surgery Type**	
Chest Surgery	177 (86.8%)
Stage 1 Vaginectomy	9 (4.4%)
Stage 2 Phalloplasty	9 (4.4%)
Combined Stage 1 Vaginectomy and Stage 2 Phalloplasty	3 (1.5%)
Vaginoplasty	5 (2.5%)
Combined Stage 1 Vaginectomy and Metoidioplasty	1 (0.5%)

**Table 2:** Chest Surgeries in the Three-Year Case Series at the Center for Gender Surgery (2017–2020).

Variable	Median (Range)or n (%)
Number of Chest Reconstruction Cases	177
*Demographics*	
Age (years)	18 (15, 33)
Patient a Minor on Date of Surgery	65 (36.7%)
Weight (kg)	69.3 (44.3, 141.4)
**ASA-PS**	
I	40 (22.6%)
II	128 (72.3%)
III	9 (5.1%)
**Gender Identity**	
Trans Man	163 (92.1%)
Trans Woman	5 (2.8%)
Non-binary	8 (4.5%)
Cis Female	1 (0.5%)
*Perioperative Characteristics*	
**Discharge Plan**	
Home	33 (18.6%)
Floor	144 (81.4%)
ICU	0 (0%)
**ICU Admission Planned**	
**Adverse Events**	8 (4.5%)
Hematoma	7 (4%)
Airway Adverse Event	1 (0.5%)
Readmission 48 h–30 days	5 (2.8%)
Reason for Readmission	Hematoma
Hospital Length of Stay (days)	1.1 (0.2, 5.3)
*PACU Data*	
PACU Emesis	2 (1.1%)
PACU Pain Score *	
Low (0–3)	112/174 (64.4%)
Medium (4–6)	52/174 (29.9%)
High (7–10)	10/174 (5.8%)
*Inpatient Data*	
Inpatient 24-h Opioid Equivalent of Morphine (mg/kg)	0.4 (0.05, 1.61)
Inpatient PONV	20 (11.3%)

ASA-PS, American Society of Anesthesiologists Physical Status; PACU, Post-Anesthesia Care Unit; PONV, Postoperative Nausea and Vomiting. * PACU Pain Scores: 3 cases “unable to answer”.

**Table 3:** Genital Surgeries in the Three-Year Case Series at the Center for Gender Surgery (2017–2020).

Variable	Stage 1 Vaginectomy	Stage 2 Phalloplasty	Combined Stage 1 Vaginectomy & Stage 2 Phalloplasty	Vaginoplasty	Combined Stage 1 Vaginectomy & Metoidioplasty
Number of Cases	9	9	3	5	1
*Demographics*					
Age (years)	25 (22, 34)	25 (22, 34)	26 (24, 30)	19 (18, 21)	20
Patient a Minor on Date of Surgery	0 (0%)	0 (0%)	0 (0%)	0 (0%)	0 (0%)
Weight (kg)	80.8 (63.3, 102)	80.2 (63.7, 102)	61.6 (54.2, 96.2)	53.9 (50.3, 70.1)	64.5
**ASA-PS**					
I	3 (33.3%)	3 (33.3%)	0 (0%)	0 (0%)	0 (0%)
II	6 (66.7%)	6 (66.7%)	3 (100%)	4 (80%)	1 (100%)
III	0 (0%)	0 (0%)	0 (0%)	1 (20%)	0 (0%)
**Gender Identity**					
Trans Man	9 (100%)	9 (100%)	3 (100%)	0 (0%)	0 (0%)
Trans Woman	0 (0%)	0 (0%)	0 (0%)	5 (100%)	1 (100%)
Non-binary	0 (0%)	0 (0%)	0 (0%)	0 (0%)	0 (0%)
Cis Female	0 (0%)	0 (0%)	0 (0%)	0 (0%)	0 (0%)
*Perioperative Characteristics*					
**Discharge Plan**					
Home	0 (0%)	0 (0%)	0 (0%)	0 (0%)	0 (0%)
Floor	9 (100%)	2 (22.2%)	0 (0%)	5 (100%)	1 (100%)
ICU	0 (0%)	7 (77.8%)	3 (100%)	0 (0%)	0 (0%)
ICU Admission Planned	N/A	7/7 (100%)	3 (100%)	N/A	N/A
Adverse Events	0 (0%)	0 (0%)	0 (0%)	0 (0%)	0 (0%)
Readmission 48 h–30 days	0 (0%)	0 (0%)	2 (66.7%)	1 (20%)	0 (0%)
Reason for Readmission	N/A	N/A	Arm Pain Concerns; Uncontrolled Pain	Pain	N/A
*PACU Data*					
PACU Emesis	0 (0%)	N/A	N/A	0 (0%)	0 (0%)
**PACU Pain Score**					
Low (0–3)	3 (33.3%)	N/A	N/A	5 (100%)	1 (100%)
Medium (4–6)	2 (22.2%)	N/A	N/A	0 (0%)	0 (0%)
High (7–10)	4 (44.4%)	N/A	N/A	0 (0%)	0 (0%)
*Inpatient Data*					
Inpatient 24-h Opioid Equivalent of Morphine (mg/kg)	0.53 (0.06, 1.53)	1.5 (0.54, 3.42)	0.57 (0.15, 0.99)	0.09 (0.06, 0.24)	
Inpatient PONV	1 (11.1%)	0 (0%)	0 (0%)	0 (0%)	0 (0%)

Continuous data are presented as median (range) and categorical data are presented as n (%). ASA-PS, American Society of Anesthesiologists Physical Status; PACU, Post-Anesthesia Care Unit; PONV, Postoperative Nausea and Vomiting; N/A, not applicable.

**Table 4:** Perioperative Complexities Unique to Gender-Diverse Youth identified by Anesthesia Providers.

Self-Identification and Terminology	As a teaching hospital, new anesthesia trainees, nurses, and staff are caring for an increasing number of transgender patients. The lack of a proper identification process results in providers calling patients by their wrong name and pronouns. Unfamiliar terminology results in misgendering, increased anxiety, or emotional distress for gender-diverse youth in the perioperative environment [9].
Gender-Identity Fields	Gender-diverse youth arrive at the preoperative visit with inconsistent forms, insurance cards, and paperwork, which puts them at risk of misgendering and other intentional and unintentional microaggressions [9]. Inconsistencies in the electronic medical record may also increase the risk of medical error [10].
Past Medical History and Chronic Conditions	In addition to mental and behavioral issues, many transgender patients coming for surgical procedures presented with coexisting morbidities, which affected surgical risk [7]. Transgender youth with complex medical and surgical histories presenting for same-day procedures challenged anesthesia providers in providing the highest standard of care with limited resources.
Perioperative Testing and Planning	Patients sometimes faced unnecessary laboratory testing prior to surgery. Unclear identification of the sex assigned at birth, gender identity, and presence or absence of a uterus resulted inappropriate perioperative HCG testing, which caused distress to patients and families [9]. With a number of patients on puberty blockers, menstrual suppression, and/or cross-sex hormone therapy, anesthesia providers questioned medication interactions, thromboembolic prophylaxis, and the need for additional laboratory testing.
Psychosocial Issues	Physical, emotional issues, and support systems needed to be addressed prior to surgery [9]. Before top or bottom surgery, patients must have met World Professional Association of Transgender Health (WPATH) standard of care eligibility criteria and obtained well-documented mental health screenings [5]. The experience of being transgender in a society that does not accept or affirm one’s identity results in an increased risk of a number of behavioral health concerns: including anxiety, depression, substance abuse, trauma, and suicidality [11,12,13,14].

**Table 5:** GASPP Initiatives.

1. Individualized Anesthesia Management Guidelines	Mastectomy, sometimes referred to as “top surgery” is an important step for female-to-male (FTM) transgender patients. The goal of the surgery is to remove breast tissue and create a masculine chest contour [7]. Anesthesia considerations focus on preoperative anxiety, and a balanced intravenous technique to minimize postoperative emesis. Surgical considerations require baseline awake blood pressures to assess hemostasis and use of tranexamic acid to reduce postoperative bleeding.
	Phalloplasty procedures consist of several steps, including vaginectomy, urethral lengthening, scrotoplasty, and creation of the neophallus, and phalloplasty is performed with a free tissue transfer from the radial forearm [7]. Communication between the anesthesia and surgery team is important to optimize the success of the vascular anastomosis, other concerns are shared with metoidioplasty (below).
	Metoidioplasty is the creation of a phallus (penis) from the hormonally-enlarged clitoris with the goal to stand and urinate, and usually also includes vaginectomy [7]. The anesthesia considerations for both phalloplasty and metoidioplasty focus on preoperative anxiety, fluid management, pain management strategies, and positioning as extended operating times present with an increased risk of peripheral neuropathies to both upper and lower extremities.
	Vaginoplasty involves the creation of a sensate clitoris from the penile glans, an aesthetic vulva using scrotal tissue, and (usually) a neovaginal canal [7]. Many techniques are used in the creation of the neovaginal canal. The most common technique for vaginoplasty is penile inversion, in which the penile and scrotal skin is inverted to form the lining of the vaginal canal [7]. Other options include bowel and peritoneal tissues. The anesthesia considerations for penile inversion vaginoplasty focus on preoperative anxiety, pain management (epidural anesthesia), hemostasis, and positioning as extended operating times present an increased risk of peripheral neuropathies to both upper and lower extremities.
2. Anesthesia Scheduling	Once a chest or reconstructive genital procedure is scheduled for surgery, a GASPP MD/CRNA is assigned to the case with preference for continuity of care. Scheduling and consistency are managed by the GASPP administrative lead and CRNA team by ICD-10-codes.
3. Direct Care Coordination	GASPP members assigned to cases call patients and contact multidisciplinary team members for perioperative planning. At the request of patients, a phone or zoom call is used to alleviate anxiety and place a familiar face on the day of surgery. An official GASPP group distribution email address was created to offer clinicians, patients, and their families a direct communication with the anesthesia specialty team.
4. Continuity of Care Program	Continuity of care for gender affirming and non-gender affirming procedures is the foundation of the program. Transgender youth who require general anesthesia for non-gender affirming procedures can access the GASPP team for perioperative assistance. The ability for patients and families to have familiar anesthesia providers helps to mitigate anxiety and risk for errors.
5. Advancing Transgender Education	Gender-diverse education is offered for anesthesia, surgical, and nursing staff on various topics, including active and passive suicide, hormonal medications, and gender affirming surgical procedures. GASPP team members mentor residents, fellows, and student registered nurse anesthetists on affirming care and current trends in gender affirming surgical techniques and anesthesia management.

## Data Availability

Not applicable.

## Appendix A

**Table A1:** Glossary of Terms.

Cisgender—an adjective describing someone whose gender identity is what would be expected for their assigned sex at birth
Gender-affirming surgery—procedures used to align an individual’s body to their gender identity, such as those used to alter primary and secondary sexual characteristics
Gender Binary—the idea that all individuals are male or female
Gender dysphoria—distress or discomfort associated with the experience of having a gender identity that does not match one’s physical body and/or the way one is perceived by society
Gender expression—the way that a person presents themselves in a gendered fashion, including clothing and hair choices, language use, etc.
Gender Identity—a person’s internal sense of themselves as male, female, non-binary, agender, or a different gender
Gender Non-Conforming—a person whose gender expression is not what would be expected for their assigned sex at birth and/or their gender identity
Non-binary—an umbrella term for people whose gender identity is neither male nor female. They may fall somewhere on the spectrum between male and female or have another gender entirely
Transgender—an adjective describing someone whose gender identity is not what would be expected for their assigned sex at birth
Transmasculine—a person assigned female at birth with a more masculine gender identity—includes transgender men as well as non-binary individuals
Transfeminine—a person assigned male at birth with a more female gender identity, includes transgender women as well as non-binary individuals

## References

[1] Herman J.L., Flores A.R., Brown T.N.T., Wilson B.D.M., Conron K.J. (2017). Age of Individuals Who Identify as Transgender in the United States.

[2] Boskey E.R., Jolly D., Tabaac A.R., Ganor O. (2020). Behavioral Health Concerns and Eligibility Factors Among Adolescents and Young Adults Seeking Gender-Affirming Masculinizing Top Surgery. LGBT Health.

[3] Mahfouda S., Moore J.K., Siafarikas A., Hewitt T., Ganti U., Lin A., Zepf F.D. (2019). Gender-Affirming Hormones and Surgery in Transgender Children and Adolescents. Lancet Diabetes Endocrinol..

[4] Boskey E.R., Johnson J.A., Harrison C., Marron J.M., Abecassis L., Scobie-Carroll A., Willard J., Diamond D.A., Taghinia A.H., Ganor O. (2019). Ethical Issues Considered When Establishing a Pediatrics Gender Surgery Center. Pediatrics.

[5] WPATH (2011). World Professional Association for Transgender Health Standards of Care for the Health of Transsexual, Transgender, and Gender Nonconforming People, 7th ed. https://www.wpath.org/publications/soc.

[6] Lane M., Ives G.C., Sluiter E.C., Waljee J.F., Yao T.-H., Hu H.M., Kuzon W.M. (2018). Trends in Gender-Affirming Surgery in Insured Patients in the United States. Plast. Reconstr. Surg. Glob. Open.

[7] Oles N., Ganor O., Aquino N.J., Boskey E.R. (2021). Surgical Affirmation for Gender-Diverse Youth. J. Pediatric Surg. Nurs..

[8] Olson-Kennedy J., Warus J., Okonta V., Belzer M., Clark L.F. (2018). Chest Reconstruction and Chest Dysphoria in Transmasculine Minors and Young Adults: Comparisons of Nonsurgical and Postsurgical Cohorts. JAMA Pediatr..

[9] Aquino N.J., Ganor O., Chrisos H.A., Oles N., Boskey E.R. (2021). Perioperative Issues With Gender-Diverse Youth. J. Pediatr. Surg. Nurs..

[10] Just B.H., Marc D., Munns M., Sandefer R. (2016). Why Patient Matching Is a Challenge: Research on Master Patient Index (MPI) Data Discrepancies in Key Identifying Fields. Perspect. Health Inf. Manag..

[11] Crissman H.P., Stroumsa D., Kobernik E.K., Berger M.B. (2019). Gender and Frequent Mental Distress: Comparing Transgender and Non-Transgender Individuals’ Self-Rated Mental Health. J. Womens Health.

[12] Hanna B., Desai R., Parekh T., Guirguis E., Kumar G., Sachdeva R. (2019). Psychiatric Disorders in the U.S. Transgender Population. Ann. Epidemiol..

[13] Lipson S.K., Raifman J., Abelson S., Reisner S.L. (2019). Gender Minority Mental Health in the U.S.: Results of a National Survey on College Campuses. Am. J. Prev. Med..

[14] Reisner S.L., Hughto J.M.W. (2019). Comparing the Health of Non-Binary and Binary Transgender Adults in a Statewide Non-Probability Sample. PLoS ONE.

[15] Ferrari L.R. (2017). How Can the Perioperative Surgical Home Be Applied to Pediatric Anesthesia Practice?. Pediatr. Anaesth..

[16] McIntosh C.A., Eckstrand K., Ehrenfeld J.M. (2016). Interdisciplinary Care for Transgender Patients. Lesbian, Gay, Bisexual, and Transgender Healthcare.

[17] Offodile A.C., Gu C., Boukovalas S., Coroneos C.J., Chatterjee A., Largo R.D., Butler C. (2019). Enhanced Recovery after Surgery (ERAS) Pathways in Breast Reconstruction: Systematic Review and Meta-Analysis of the Literature. Breast Cancer Res. Treat..

[18] Kääriäinen M., Salonen K., Helminen M., Karhunen-Enckell U. (2017). Chest-Wall Contouring Surgery in Female-to-Male Transgender Patients: A One-Center Retrospective Analysis of Applied Surgical Techniques and Results. Scand. J. Surg..

[19] Persing S., Manahan M., Rosson G. (2020). Enhanced Recovery After Surgery Pathways in Breast Reconstruction. Clin. Plast. Surg..

[20] Kimberly L.L., Folkers K.M., Friesen P., Sultan D., Quinn G.P., Bateman-House A., Parent B., Konnoth C., Janssen A., Shah L.D. (2018). Ethical Issues in Gender-Affirming Care for Youth. Pediatrics.

[21] Rider G.N., McMorris B.J., Gower A.L., Coleman E., Eisenberg M.E. (2018). Health and Care Utilization of Transgender and Gender Nonconforming Youth: A Population-Based Study. Pediatrics.

[22] James S.E., Herman J.L., Rankin S., Keisling M., Mottet M., Anafi M. (2016). The Report of the 2015 U.S. Transgender Survey.

[23] Lindqvist E.K., Sigurjonsson H., Möllermark C., Rinder J., Farnebo F., Lundgren T.K. (2017). Quality of Life Improves Early after Gender Reassignment Surgery in Transgender Women. Eur. J. Plast. Surg..

[24] Passos T.S., Teixeira M.S., Almeida-Santos M.A. (2020). Quality of Life After Gender Affirmation Surgery: A Systematic Review and Network Meta-Analysis. Sex. Res. Soc. Policy.

[25] Andréasson M., Georgas K., Elander A., Selvaggi G. (2018). Patient-Reported Outcome Measures Used in Gender Confirmation Surgery: A Systematic Review. Plast. Reconstr. Surg..

[26] Barone M., Cogliandro A., Di Stefano N., Tambone V., Persichetti P. (2017). A Systematic Review of Patient-Reported Outcome Measures Following Transsexual Surgery. Aesth. Plast. Surg..

[27] Dy G.W., Nolan I.T., Hotaling J., Myers J.B. (2019). Patient Reported Outcome Measures and Quality of Life Assessment in Genital Gender Confirming Surgery. Transl. Androl. Urol..

[28] Klassen A.F., Kaur M., Johnson N., Kreukels B.P., McEvenue G., Morrison S.D., Mullender M.G., Poulsen L., Ozer M., Rowe W. (2018). International Phase I Study Protocol to Develop a Patient-Reported Outcome Measure for Adolescents and Adults Receiving Gender-Affirming Treatments (the GENDER-Q). BMJ Open.

[29] Kattari S.K., Walls N.E., Speer S.R., Kattari L. (2016). Exploring the Relationship between Transgender-Inclusive Providers and Mental Health Outcomes among Transgender/Gender Variant People. Soc. Work. Health Care.

